# The Royal Society of Medicine

**Published:** 1912-10-19

**Authors:** 


### SOME INTERESTING CASES.

### The Royal Society of Medicine.

At the meeting of the Clinical Section on Friday, 11th inst., under the presidency of Sir William Osler, several cases of considerable interest were shown and discussed.

Mr. Morriston Davis showed a man, æt. forty-seven, who had had syphilis and was a heavy drinker. Sixteen months ago a toe became gangrenous and was amputated, the wound remaining open. Five months later two patches of gangrene appeared on other toes, and there was coldness and discolouration of the foot generally. The amputation wound still remained unhealed. Therefore an arteriovenous anastomosis was performed in Hunter's canal in August 1911, the proximal end of the superficial femoral artery being united to the peripheral end of the femoral vein, and the proximal end of the vein and the distal end of the artery being ligatured. The pain and discolouration disappeared after the operation, the foot became warm, the gangrenous patches separated off, and the wound of the amputation healed. The patient began to walk a month after the operation, and has now been able for some months to walk with the aid of a stick. There is no pain, discolouration, nor œdema.

A Boy with Dermatolysis.

Dr. H. Batty Shaw and Mr. Percy Hopkins exhibited a boy, æt. seven, the subject of double-jointedness, dermatolysis (or "elastic skin"), showing a great tendency to bruising, and with a large number of subcutaneous tumours. At birth (in the eighth month) his bones seemed very soft and "jelly-like," and he was said to look like a little Chinaman. His capacity to walk was delayed, and his walking was not yet satisfactory. The mother stated that the anterior fontanelle did not close until the fifth year. There is marked lateral curvature of the dorsal spine, with convexity to the left, but an $x$-ray examination showed that the curvature was independent of any deformity of the vertebræ. Dr. James Galloway, discussing the case, referred to the well-known case which paraded various London hospitals and went by the name of "the elastic-skin man." In Berlin he parted with a portion of his skin for the purpose of investigation, and the elastic tissue in it was normal, though the fibres were very fine and tended to be granular. The tumours seemed to resemble fibromata, but consisting of elastic tissue instead of fibrous tissue.

### The Cervical Rib.

Sir William Osler showed a girl, æt. twenty, with cervical rib, in whom there was a remarkable circulatory disturbance, probably in association with it. She was a healthy girl, with a good family history, by profession a teacher. There was no enlargement of glands, but the right pupil was a little larger than the left. Five months ago she noticed that after using the right arm it became darker in colour, the hand swelled, and the veins in the neck became distended. After using the muscles of the right hand for a minute or two the skin became red, at first on the inner side above the elbow; then the redness became general, and the arm measured one-quarter more than before the exertion commenced. The pulse in the right radial became smaller, the blood pressure fell from 115 mm. to 90 mm., the veins in the neck, particularly the external jugular, became enlarged, and there was a prominent venous swelling above the inner end of the clavicle. Continuation of the exertion resulted in the arm feeling numb and dead, and she had to rest. Skiagraphy revealed a moderate-sized cervical rib on each side, but there might be cartilaginous extensions. In some cases of cervical rib the symptoms were suggestive of Raynaud's disease. He proposed removal of the cervical ribs.

### A Notable Case.

A series of interesting surgical cases by Mr. Laurie McGavin included one of abdomino-perineal excision of the rectum, done sixteen months ago, in which transverse colostomy was performed under spinal analgesia and chloroform. The patient was a woman, æt. sixty-seven, and had carcinoma of the first and second parts of the rectum, with signs of commencing obstruction. Six centigrams of stovaine-glucose were injected spinally, and the operation was as follows: transverse colostomy by Maydl's method; laparotomy, ligature of both internal iliac arteries; sigmoid double ligatured, divided, and ends inverted by Lambert's sutures. Bowel with meso-sigmoid and glands stripped down to pelvis. At this point light chloroform anæsthesia was given for half an hour, but the patient was never quite unconscious. The chloroform was then stopped and the patient placed in the lithotomy position. The anus was closed by suture, and the whole length of rectum and lower part of sigmoid were removed with growth intact. The patient was fully conscious during the last twenty minutes of the operation, and left the theatre with a pulse of 85, and there was no sign of shock.

